# Development of Palladium and Platinum Decorated Granulated Carbon Nanocomposites for Catalytic Chlorate Elimination

**DOI:** 10.3390/ijms231810514

**Published:** 2022-09-10

**Authors:** Emőke Sikora, Gábor Muránszky, Ferenc Kristály, Béla Fiser, László Farkas, Béla Viskolcz, László Vanyorek

**Affiliations:** 1 Institute of Chemistry, University of Miskolc, H-3515 Miskolc, Hungary; 2 Institute of Mineralogy and Geology, University of Miskolc, H-3515 Miskolc, Hungary; 3 Higher Education and Industrial Cooperation Centre, University of Miskolc, H-3515 Miskolc, Hungary; 4 Ferenc Rakoczi II Transcarpathian Hungarian College of Higher Education, 90200 Beregszász, Transcarpathia, Ukraine; 5 BorsodChem Ltd., Bolyai tér 1., H-3700 Kazincbarcika, Hungary

**Keywords:** GCNC, N-BCNT, hydrogenation, gel beads, sodium alginate

## Abstract

Granulated carbon nanotube-supported palladium and platinum-containing catalysts were developed. By using these, remarkable catalytic activity was achieved in chlorate ion hydrogenation. Nitrogen-doped bamboo-like carbon nanotubes (N-BCNTs) loaded gel beads were prepared by using Ca^2+^, Ni^2+^ or Fe^3+^ ions as precursors for cross-linking of sodium alginate. The gel beads were carbonized at 800 °C, and these granulated carbon nanocomposites (GCNC) were used as supports to prepare palladium and platinum-containing catalysts. All in all, three catalysts were developed and, in each case, >99 n/n% chlorate conversion was reached in the aqueous phase by using the Pd-Pt containing GCNCs, moreover, these systems retained their catalytic activity even after repeated use.

## 1. Introduction

Carbon has one of the most versatile allotropic modifications on Earth. From powder to fibers, there is a wide range of varieties, including activated carbon, fullerenes, carbon nanotubes, carbon fibers, graphene and of course diamond. These carbon variants provide special electrical, mechanical, and optical properties that led to previously unimaginable applications including catalysis [1,2,3,4,5,6]. Carbon-based materials play an exceptional role in the development of catalytic systems.

To achieve an environmentally friendly chemical industry, catalytic processes are essential and need to be applied as widely as possible. Therefore, to increase the catalytic activity and selectivity of the catalysts and to reduce the cost of catalyst production, the necessary heat and amount of chemicals are inevitable [7]. In parallel with the development of new active phases, intensive research is being carried out by both academic and industrial groups to develop new catalyst supports that can modify the catalytic activity and selectivity of existing active phases [8,9,10,11,12,13]. Besides cost-effectiveness, carbonaceous solid catalysts have the unique ability of keeping their activity not only in organic solutions but also in aqueous solutions [14]. It was revealed that heat treatment can improve the oxidation resistance of porous graphene more efficiently than conventional porous carbon [6]. These unique properties make this material promising catalyst support for catalytic reactions conducted under oxidative conditions [6].

There are several examples in the literature where carbon-based catalysts have been used for halogenate reduction by catalytic hydrogenation [15,16,17,18]. Nitrogen-doped bamboo-like carbon nanotubes (N-BCNTs) and non-doped multi-walled carbon nanotubes were compared in terms of their activity and the former was better because electron-rich N atoms promote electron transfer processes [19,20]. N-BCNTs are special types of carbon nanotubes, made from nitrogen-containing carbon compounds. Due to the nitrogen-doping, defect sites are also appearing in the structure of the nanotubes, which are potential binding sites for the catalytically active metal particles [21].

The drawback of the most promising carbon forms is that they often fall into the nano range in more dimensions, which makes their use difficult on an industrial scale (especially in fixed-bed catalytic reactions) and, last but not least, makes them dangerous to handle (e.g., inhalation). Consequently, it is important to search for and develop methods that allow the large-scale synthesis of carbon nanostructures and their direct macroscopic sizing for immobilization [6,22,23,24,25]. Structured catalyst support with oxygenated functional groups from commercial graphite felt (OFG) raw material was prepared and applied in Pd/OFG catalyst synthesis, which was tested through the liquid-phase hydrogenation of cinnamaldehyde and it was found that the system expressed excellent catalytic activity, and stability as well as recyclability [26]. Furthermore, monolithic biochar-based catalysts decorated with graphitic carbon-covered metal nanoparticles were synthesized [27]. This catalyst with the incorporation of Ni-Co alloy nanoparticles achieved a synergistic effect of Co and Ni nanoparticles in tar decomposition, exhibiting higher activity and better stability than the catalyst incorporating pure Co and Ni nanoparticles for both toluene cracking and steam reforming processes [27]. Nanodiamond-based monolith within an N-doped mesoporous carbon matrix was prepared [28]. Cheap food-grade components were used as glue for the dispersion of nanodiamonds and this metal-free composite system served as a highly stable and well-performing catalyst for the conversion of ethylbenzene to styrene [28]. It has also been found that the incorporated nitrogen in this monolith contributes to increased styrene selectivity [28]. The use of activated carbon cloth as platinum catalyst support has also been investigated and used in catalytic hydrogenation [29]. In addition to their activity and the particular shape of the cloth support, the possibility of being used in a wide range of temperatures is very advantageous [29]. Granulated nitrogen-doped graphene oxide aerogels (N-dGOA) were synthesized and showed encouraging electro-catalytic activity in oxygen reduction, which makes them applicable in areas such as hydrogen and thermal energy storage [30]. In addition, granulated platinum-decorated carbon nanotubes were found to have much better catalytic activity in the liquid phase hydrogenation of nitrobenzene than their platinum-decorated activated carbon (AC) counterparts [31]. The granulated CNTs had larger pores than the AC particles, which gave a faster mass transfer rate of H_2_ and helped to produce aniline with high selectivity [31]. Nitrogen-doped carbon nanotubes (N-CNT) supported on a macroscopic structure of SiC have shown to be an active and selective metal-free catalyst for the low-temperature oxidation of H_2_S into elemental sulfur [22]. The macroscopic shaping allows us to avoid the problems with the handling and transport of the nanoscopic system, and this hybrid metal-free catalyst with controlled macroscopic shape can be efficiently employed in a fixed-bed configuration without facing the problem with the pressure drop across the catalytic bed [22].

To combine the above-mentioned positive effects of carbon-based materials, N-BCNT was used to prepare a granulated catalyst support and by using it sodium alginate nanotube-loaded gel beads were created. These beads were carbonized and used as supports to develop palladium and platinum catalysts and tested in catalytic chlorate hydrogenation.

## 2. Materials and Methods

### 2.1. Materials

For the CCVD synthesis of N-BCNTs, *n*-butylamine (Sigma Aldrich, St. Louis, MO, USA) was used as a carbon source and nickel(II) nitrate hexahydrate (Ni(NO_3_)_2_ · 6H_2_O, ThermoFisher GmbH, 76870 Kandel, Germany) was applied as a catalyst on magnesium oxide (Sigma Aldrich). Nitrogen (99.995%) was used as carrier gas (Messer Hungary Ltd, Budapest, Hungary). Sodium alginate (Sigma-Aldrich, 3050 Saint Louis, MO 63103, USA) was applied to prepare the N-BCNT gelatine beads along with calcium-chloride (CaCl_2_ · 2H_2_O, Merck Ltd, Darmstadt, Germany), nickel(II) nitrate hexahydrate (Ni(NO_3_)_2_ · 6H_2_O, ThermoFisher GmbH, 76870 Kandel, Germany), and iron(III) nitrate nonahydrate (Fe(NO_3_)_3_ · 9H_2_O, VWR Int. Ltd., B-3001 Leuven, Belgium). To prepare the final catalyst, palladium(II) nitrate dihydrate (Pd(NO_3_)_2_ · 2H_2_O, Merck Ltd.) and hexachloroplatinic acid (H_2_PtCl_6_, Merck Ltd.) were applied as Pd and Pt precursors, respectively. During the chlorate hydrogenation tests, potassium iodide (KI, Merck), 35 wt% hydrochloric acid (HCl, VWR), and potassium chlorate (KClO_3_) were applied.

### 2.2. Characterization Techniques

High-resolution transmission electron microscopy (HRTEM, FEI Technai G2 electron microscope, 200 kV) was used to characterize the nanoparticles. The sample preparation was carried out by using the aqueous suspension of the nanoparticles, which was dropped on 300 mesh copper grids (Ted Pella Inc, Redding, CA, USA). The surface of the samples was further examined by a Helios G4 PFIB CXe Plasma Focused Ion Beam Scanning Electron Microscope (PFIB-SEM) equipped with an EDAX Octane Elect EDS System and APEX Analysis Software. Carbon tape was used for sample preparation. EDS maps were created with a 1024 × 800 resolution, and 1 frame was recorded with a 1000 μs collecting time. The particle diameters of the nanoparticles were manually scaled using the ImageJ program, based on the scale bar of SEM images. The qualitative and quantitative analysis of the different metals and metal-oxide forms was carried out by using X-ray diffraction (XRD) measurements with Rietveld analysis. Bruker D8 diffractometer (Cu-Kα source) in parallel beam geometry (Göbel mirror) with Vantec detector was applied. The metal (palladium and platinum) content of the catalyst samples was determined by using a Varian 720 ES inductively coupled optical emission spectrometer (ICP-OES). For the ICP-OES measurements, the samples were burned in air, after the remaining ash was solved in aqua regia. The specific surface area of the catalysts was also measured by CO_2_ adsorption experiments using Micromeritics ASAP 2020 sorptometer and the calculations were carried out based on the Dubinin-Ashtakov isotherm. The types of the incorporated nitrogen atoms were determined by X-ray photoelectron spectroscopy (XPS), using a Kratos XSAM-800 XPS instrument. The MgKα X-ray source was operated with 120 W (12 kV, 10 mA). Samples were examined on double-sided carbon tape. Survey spectra were collected with a pass energy of 80 eV and 1 eV step size. The N-BCNTs were examined by using Raman microscopy (WITECH 3112973 instrument with HeNe laser, λ = 632.92 nm). The structural defects were quantified by calculating the ratio of the intensities of the defect peak (D-peak, ~1340 cm^−1^) and the graphite peak (G-peak, ~1580 cm^−1^) (ID/IG).

### 2.3. Synthesis of the Nitrogen-Doped Bamboo-Like Carbon Nanotubes (N-BCNTs)

N-BCNTs were synthesized from *n*-butylamine by using the catalytic chemical vapor deposition (CCVD) method in a quartz reactor, which was placed in a tube furnace. Butylamine was dosed (16.2 mL h^−1^) into the quartz reactor with a syringe pump. The carrier gas was nitrogen (100 scm), and the carbon nanotubes were synthesized at 750 °C for 20 min by using of 5 wt% nickel-containing MgO catalyst (2.5 g). After the N-BCNT synthesis, the catalyst was removed by cc. hydrochloric acid. 

### 2.4. Preparation of Catalyst Supports and Pd-Pt Containing Catalysts

The preparation of the catalyst supports is similar to a method applied in our previous work, with some modifications [32]. A mixture of 100 mL distilled water, 0.75 g sodium alginate, and 1 g N-BCNT were prepared by using a Hielscher homogenizer. This mixture was added dropwise to 300 mL 5.5 g CaCl_2_ or 7.5 g Ni(NO_3_)_2_ or 10.86 g Fe(NO_3_)_3_ solution by using a syringe pump. After the preparation, the remaining solution was removed and the spheres were washed with distilled water and then, dried at 370 K for 24 h. The prepared beads were calcinated at 800 °C under a nitrogen flow for 60 min. For the calcium-containing samples, a concentrated hydrochloric acid wash was also applied prior to heat treatment. All in all, three different GCNC (granulated carbon nanocomposite) supports were prepared.

Palladium and platinum were added to the supports by using an impregnation method. A total of 1.15 g catalyst support was added to a 50 mL metal solution containing 0.1 g of Pd(NO_3_)_2_ · 2 H_2_O and 0.01 g of H_2_PtCl_6_. This was followed by vacuum evaporation and reduction at 673 K in the H_2_ stream for 30 min.

### 2.5. Catalytic Hydrogenation of Chlorate Ions

The prepared catalysts were tested in catalytic chlorate hydrogenation. To test the catalytic activity of each system, potassium chlorate (200 mg/dm^3^) was hydrogenated in the presence of a 1 g catalyst in aqueous media. Gas supply was provided (40 sccm nitrogen and 100 sccm hydrogen) during the experiments and the temperature was set to 80 °C by using a Julabo circulator. The solution was placed in a side-inlet gas washing bottle with a fritted disc. The hydrogenation was carried out for 3 h in each case, and sampling took place at 0, 5, 15, 30, 45, 60, 90, 120, 150, and 180 min. Thereafter, distilled water was used to wash the catalysts which then, were dried at 105 °C overnight. UV-6300PC spectrophotometer was applied at 351 nm to determine the chlorate concentration in the collected samples. During the measurements, the following redox reaction was considered between iodide and chlorate ions:

KClO_3_ + 6 KI + 6 HCl → 3 H_2_O + 3 I_2_ + 7 KCl (1)

The intensity of the color, and thus, the absorbance of the samples changed due to iodine formation, from which the chlorate concentration can be determined by appropriate calibration. Potassium chlorate solutions with different concentrations (0, 50, 100, 150, and 200 mg/dm^3^) were prepared for calibration. A total of 100 mg potassium iodide and 1 mL HCl were added to 1 mL sample, and then, it was diluted by using 50 mL distilled water, and this was measured by the spectrophotometer. 

## 3. Results and Discussion

### 3.1. Characterization of the Catalyst Supports

**Figure 1:**
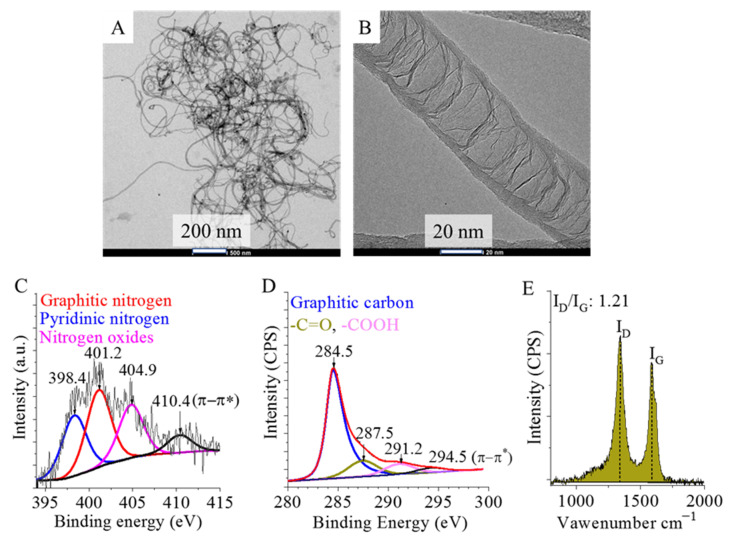
HRTEM images (**A**,**B**), deconvoluted N1s (**C**) and C1s band (**D**) on the XPS spectrum and Raman spectrum (**E**) of the synthesized nitrogen-doped bamboo-like carbon nanotubes (NBCNTs).

By using transmission electron microscopy, the fibrous structure of the prepared N-BCNTs was verified (Figure 1A). The graphene layers dividing the nanotubes into segments are also visible at higher resolution (Figure 1B). 

At the edge of the before-mentioned graphene layers, the carbon atoms are easily oxidized, and thus, several hydroxyl and carboxyl functional groups can be formed on the surface of the N-BCNTs. Due to the deprotonation of these functional groups in aqueous dispersions, negative charges appear, which lead to the decreasing electrokinetic potential of the N-BCNTs. This can be used effectively to anchor the catalytically metal ions (i.e., palladium- or nickel-ions) on the surface of the nanotubes by ion exchange adsorption and electrostatic interactions. On the other hand, this can also promote the continuous, and homogenous coverage of the surface of the nanotubes by metal nanoparticles. Moreover, the interaction between the nanoparticles and the N-BCNTs will be favored. Thus, the extraordinary bamboo-like structure formed due to the incorporation of nitrogen atoms in the graphitic structure of the nanotubes is beneficial.

On the deconvoluted N1s band of the N-BCNTs’ XPS spectrum, three peaks were identified at 398.4 eV, 401.2 eV, and 404.9 eV binding energy, which are associated with the pyridinic, and graphitic nitrogen atom types, and nitrogen oxides, respectively (Figure 1C). The C1s band of the N-BCNTs’ XPS spectrum was also deconvoluted and three peaks were identified at 284.5 eV, 287.5 eV, and 291.2 eV binding energy which can be associated with carbon atoms of the graphitic character (C=C- and -C-C- bonds), C=O, and C atoms in carboxyl functional groups.

The characteristic bamboo-like structure of the nanotubes is achieved by the nitrogen incorporation into the system. This also increases crystal lattice defects and disorder in the graphitic structure, which makes the N-BCNTs less graphitic compared to conventional non-doped multi-walled carbon nanotubes (MWCNTs). Raman spectroscopy was applied to quantify the disordered character of the structure of the nanotubes (Figure 1E). The ratio of the intensities of the defect peak (D-peak, ~1340 cm^−1^) and the graphite peak (G-peak, ~1580 cm^−1^) (ID/IG) was calculated [33]. The impurities or disorder in the nanotubes are defined by the D-peak, while the G-peak is associated with carbon-carbon bond stretching. The ID/IG ratio is relatively high, 1.21 in the case of the studied N-BCNTs. (For reference, Kim et al used heat treatment to improve the degree of CNT crystallinity, resulting a ratio of 0.4 [34]). Thus, due to the presence of lattice defects, the nitrogen-doped nanotubes are prone to modification, and various functional groups (-COOH, -OH) could occur on their surfaces which led to high dispersibility in polar solvents.

The N-BCNTs were embedded into alginate gel beads. Ca^2+^, Ni^2+^, or Fe^3+^ ions were applied as precursors for cross-linking sodium alginate, and thus, three different N-BCNT loaded gel beads (or granulated carbon nanocomposites, GCNC) were created, GCNC, Ni-GCNC, and Fe-GCNC, respectively. The advantage of transition metals compared to calcium is that they can show catalytic activity or influence the catalytic behavior of the noble metal-containing hydrogenation catalysts. The nickel and iron-containing, N-BCNT loaded alginate gel beads were carbonized at 800 °C in nitrogen flow.

**Figure 2:**
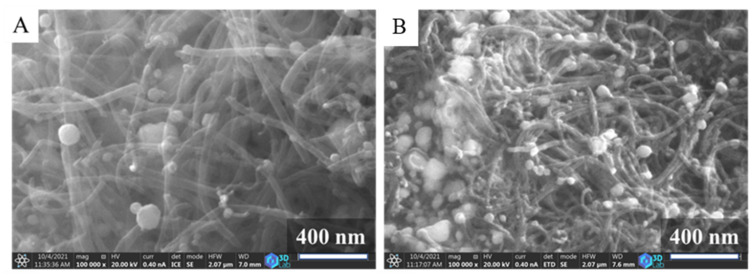
SEM images of the nickel (**A**) and magnetite (**B**) containing N-BCNT loaded gel beads.

On the SEM images of the carbonized supports (Supplementary Materials Figure S1A,B), the fibrous structure of the N-BCNTs is visible and decorated with spherical objects, which are nickel nanoparticles that remained from the CCVD synthesis. More nanoparticles are visible on the SEM images of the Ni- and Fe-GCNC samples (Figure 2A,B) which contain not only the residual Ni from the CCVD synthesis, but also Ni and Fe particles that have replaced Na from Na-alginate.

**Figure 3:**
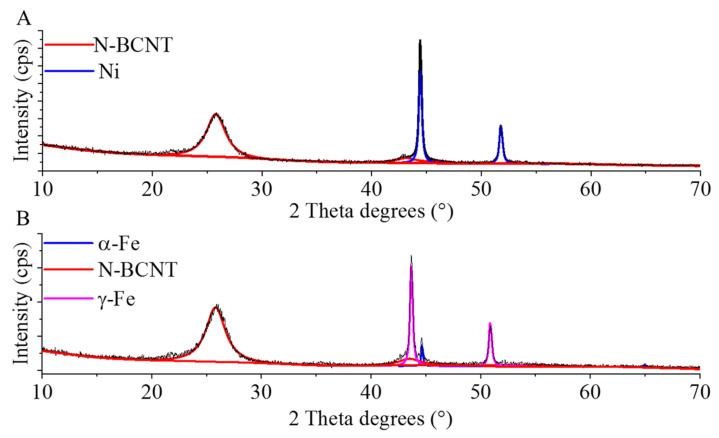
XRD pattern of the nickel (**A**) and magnetite (**B**) containing granulated carbon nanocomposites (GCNC).

To determine the composition of the nickel and iron-containing beads, XRD measurements were carried. On the diffractogram of the Ni-GCNC, peaks were identified at 44.4° and 51.8° two theta degrees, which can be associated with (111) and (200) reflexions (PDF 04-0850), and characteristic to the metallic nickel phase (Figure 3A). The reflexions at 25.8° (002) and 43.1° (100) indicate the presence of a carbon phase as well (PDF 75-1621). In the case of the Fe-GCNC sample, the (111) and (200) reflexions at 43.7° and 50.9° 2 ϴ degrees (PDF 06-0696) belong to the α-Fe phase (Figure 3B). Furthermore, the reflexion at 44.6° (110) confirmed that the carbonization of the gel beads led to the formation of a γ-Fe phase. The presence of elemental metallic particles can be explained by the reducing effect of carbon at high temperatures (800 °C).

### 3.2. Characterization of the Noble Metal Containing Catalysts

**Figure 4:**
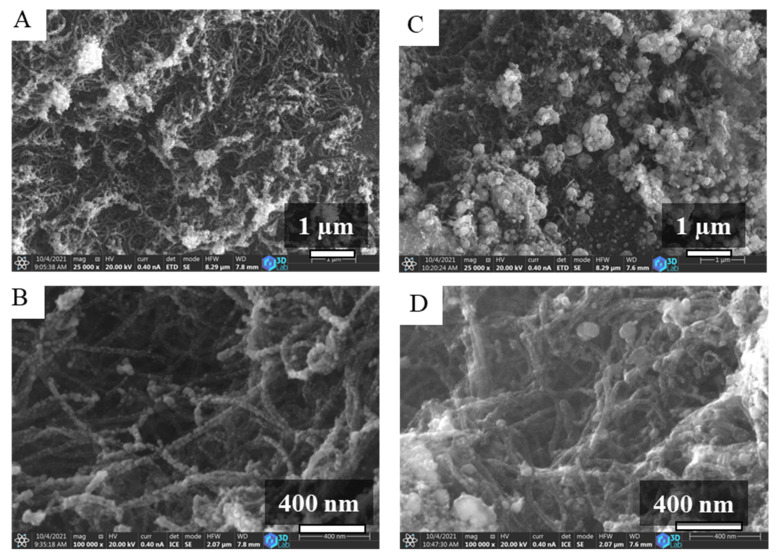
SEM images of the nickel (**A**,**B**) and magnetite (**C**,**D**) containing, palladium and platinum decorated granulated carbon nanocomposites (GCNC). Pd-Pt/Ni-GCNC—(**A**,**B**) and Pd-Pt/Fe-GCNC (**C**,**D**).

The GCNC, Ni-GCNC and Fe-GCNC supports were used for catalyst preparation, which was impregnated with the solutions of palladium and platinum precursors. The dried impregnated supports were activated in a hydrogen atmosphere at 400 °C. The surface of the grains is richly covered by metal nanoparticles in the case of the Pd-Pt/Ni-GCNC and Pd-Pt/Fe-GCNC catalysts (Figure 4A,C). A significant amount of these nanoparticles is aggregated, but the fibrous structure of the N-BCNTs is still visible at higher resolution (Figure 4B,D). The Pd-Pt/GCNC sample is similar in terms of surface coverage and aggregation (Supplementary Materials Figure S1C,D). In the case of the Pd-Pt/Fe-GCNC catalyst, the particle size of the anchored Pd and Pt crystallites on the N-BCNT surface is smaller compared to those in the case of the Pd-Pt/Fe-GCNC (Figure 4). To confirm this, the SEM images were used to prepare size distribution diagrams (Supplementary Materials Figure S2). The smallest average particle size was measured in the case of Pd-Pt/Ni-GCNC (14.4 ± 7.0 nm), while the largest value corresponds to Pd-Pt/Fe-GCNC (105.2 ± 58.1 nm) and Pd-Pt/Ca-GCNC is in the middle in terms of this property (46.5 ± 14.3 nm). The median values were 12.5 nm, 89.1 nm, and 46.5 nm for Pd-Pt/Ni-GCNC, Pd-Pt/Fe-GCNC, and Pd-Pt/Ca-GCNC, respectively. Based on the mean and standard deviation values, the nickel-containing sample was the most homogeneous with the smallest particles and the most uniform in terms of particle size.

**Figure 5:**
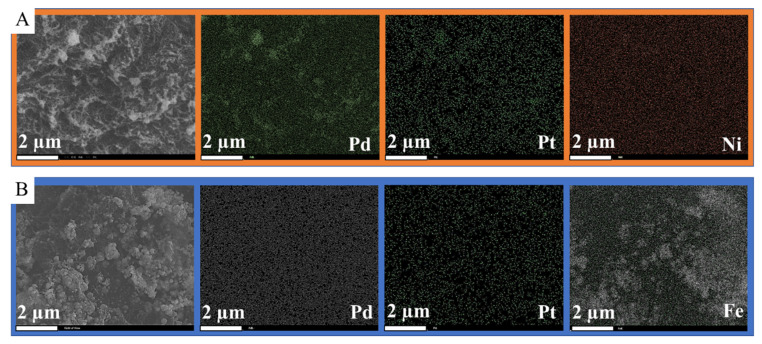
Elemental maps of the nickel (**A**) and magnetite (**B**) containing, palladium and platinum decorated granulated carbon nanocomposites (GCNC).

Elemental mapping was carried out on the Pd-Pt containing catalysts, to examine the metal distribution and coverage of the catalyst support. Elemental maps of the Pd-Pt/Ni-GCNC system indicates that the distribution of the nickel particles is homogenous and the bigger particle aggregates are mainly based on palladium, but where the Pd is enriched, Pt is also found in greater amount (Figure 5A). In the case of the Pd-Pt/Fe-GCNC catalyst, the large aggregates contain mostly iron, while the palladium and platinum nanoparticles are located in homogenous distribution on the surface (Figure 5B).

**Figure 6:**
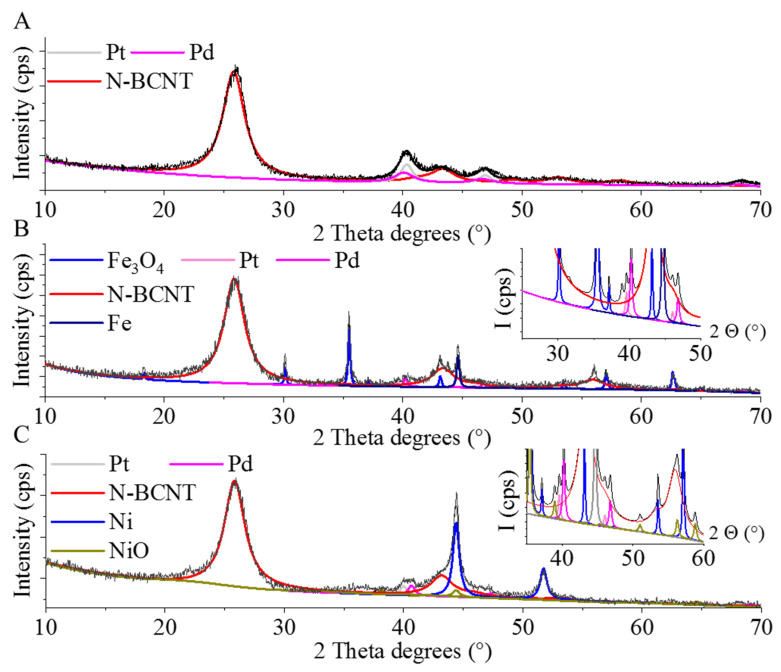
XRD patterns of the palladium and platinum decorated calcium (Pd-Pt/GCNC) (**A**), magnetite (Pd-Pt/Fe-GCNC) (**B**), and nickel (Pd-Pt/Ni-GCNC) (**C**) containing granulated carbon nanocomposites.

On the diffractogram of the iron and nickel-free Pd-Pt/GCNC catalyst, the reflexions of the palladium were identified at 40.1° (111) and 46.8° (200) two theta degrees (Figure 6A) (PDF 046–1043). The presence of elemental platinum was also verified by peaks located at 40.3 ° (111) and 46.0° (200) 2 ϴ degrees (PDF 04-0802).

In the case of the Pd-Pt/Fe-GCNC catalyst, reflexions at 18.4° (111), 30.1° (220), 35.5° (311), 43.1° (400), 53.4° (422), 57.1° (511), and 62.6° (440) two Theta degrees were located, and these can be associated with Fe_3_O_4_ (Figure 6B) (PDF 89-0691). The presence of elemental iron was also verified by the peak at 44.6° (110) two theta degrees (PDF 06-0696). The (111) and (200) reflexions of palladium and platinum are found at 40.3° and 46.7° as well as 40.1° and 46.0° two theta degrees, respectively (PDF 46-1043 and PDF 04-0802). Reflexions indicating the presence of carbon are visible at 25.8° (002) and 43.1° (100) 2 ϴ degrees (PDF 75-1621).

The presence of elemental Ni and Ni oxide in the nickel-containing system, Pd-Pt/Ni-GCNC, was verified (Figure 6C). The characteristic reflexions of Ni are found at 44.4° (111) and 51.7° (200) two theta degrees (PDF 04-0850). Peaks of the NiO phase are found at 37.2° (111) and 43.3° (200) two theta degrees (PDF 47-1049). Elemental palladium and platinum are also found in the sample.

The reductive treatment of the samples in a hydrogen atmosphere at 400 °C was enough to form palladium and platinum nanoparticles. However, despite the reductive atmosphere, due to the decomposition of the nitrate salt of palladium, nitrogen oxides were developed, which led to the formation of NiO and Fe_3_O_4_ nanoparticles. The reduction time at 400 °C was not enough to produce only the metallic form Ni and Fe, because of the difficulty to access NiO and Fe_3_O_4_ particles. Moreover, due to the palladium and platinum precursors being in hydrated form, water was present which further retards the reduction of oxides [35].

The specific surface area of the catalysts was determined before and after applying them in catalytic chlorate hydrogenation (Table 1). All in all, the surface area of the catalytic systems only slightly changed after using them five times. The largest deviation occurred in the case of the nickel-containing sample, while the smallest in the case of the Pd-Pt/Fe-GCNC.

Based on the ICP results, the GCNC sample contained 2.66 wt % Ca despite acid washing (Table 2). In the case of the Ni-GCNC and Fe-GCNC samples, ~22 wt % nickel and ~18 wt % Fe were found, respectively. All samples contain nickel, even the GCNC and Fe-GCNC supports include ~3.5 wt %, which is due to the fact, that the carbon nanotubes contain a small amount of nickel since a nickel-containing catalyst was used to synthesize the N-BCNTs. The Pd content of the noble metal-containing catalysts varied between 2.38 and 3.03 wt %, while the Pt content was between 0.28 and 0.45 wt %.

### 3.3. Comparison of the Catalytic Activity of the Developed Catalysts in Chlorate Hydrogenation

**Figure 7:**
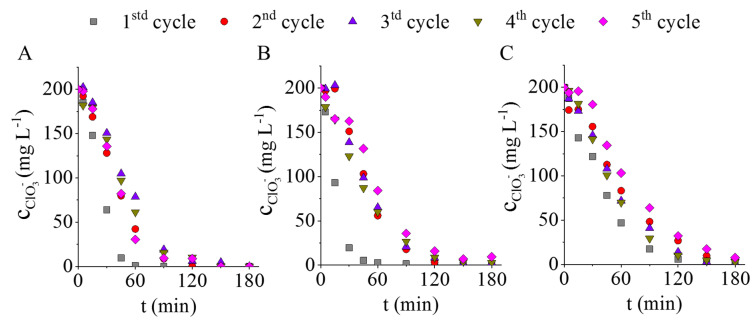
Decreasing chlorate concentration depending on the hydrogenation time by applying the developed catalysts over five cycles: Pd-Pt/Ni-GCNC (**A**), Pd-Pt/Fe-GCNC (**B**) and Pd-Pt/GCNC (**C**).

In the first experiments, the Pd-Pt/Ni-GCNC (Figure 7A) and the Pd-Pt/Fe-GCNC (Figure 7B) catalysts showed better performance, converting almost all of the chlorate to chloride in 60 min. The Pd-Pt/GCNC sample (Figure 7C) took about twice as long to achieve this level of conversion. Although some decline after the first use is experienced, significant change in the catalytic behavior of the Pd-Pt/Ni-GCNC and Pd-Pt/GCNC catalysts was not detected (Figure 7A,C). In the case of the Pd-Pt/Fe-GCNC catalyst, after the first cycle, the catalytic activity decreased to a greater extent (Figure 7B). Nonetheless, the Pd-Pt/Fe-GCNC retains this decreased catalytic activity until the end of the fifth cycle.

ICP measurements were also performed after the reuse tests (5×) of the catalysts (Table 2). The results show a significant reduction in the precious metal content after the fifth cycle in the case of the Pd-Pt/Ni-GCNC and Pd-Pt/Fe-GCNC samples. As the iron and nickel contents also significantly decreased, it is likely that the palladium and platinum crystallized on these metals have also leached from the samples’ surface during the catalytic process. This metal leaching may have caused the decrease in activity experienced during the catalytic tests (Figure 7). The Pd-Pt/GCNC sample only shows a significant decrease in its Ca content. There is no decrease in its precious metal content, but even a small increase can be seen due to sample inhomogeneity.

## 4. Conclusions

Three different catalyst supports were developed by embedding nitrogen-doped bamboo-like carbon nanotubes into alginate gel beads. Ca^2+^, Ni^2+^ or Fe^3+^ ions were applied as precursors for cross-linking of alginate. The prepared granulated carbon nanocomposites were carbonized and used as supports to create Pd and Pt-containing catalysts. All in all, three catalysts (Pd-Pt/GCNC, Pd-Pt/Ni-GCNC, and Pd-Pt/Fe-GCNC) were prepared by the impregnation method and successfully tested in chlorate hydrogenation. >99 n/n% chlorate conversion was achieved in each case. Reuse tests were also carried out, and it was found that the systems retained their activity after repeated use. Although, in the case of the nickel and iron-containing samples, a not too significant decrease in their activity was observed compared to the first cycle, which does not affect substantially their applicability. Therefore, the developed noble metal-containing catalysts are highly efficient and well applicable in chlorate hydrogenation.

## Figures and Tables

**Table 1:** Specific surface area of the developed granulated carbon nanocomposite supported Pd-Pt decorated catalysts before and after using them 5 times.

Sample	SSA (m^2^/g)
Pd-Pt/GCNC	75
Pd-Pt/GCNC 5× used	81
Pd-Pt/Ni-GCNC	214
Pd-Pt/Ni-GCNC 5× used	192
Pd-Pt/Fe-GCNC	177
Pd-Pt/Fe-GCNC 5× used	179

**Table 2:** Quantitative analysis of the granulated carbon nanocomposite supported Pd-Pt decorated catalysts based on ICP-OES measurements.

	Weight %				
	Ca	Fe	Ni	Pd	Pt
GCNC	2.66 ± 0.22	-	3.87 ± 0.21	-	-
Ni-GCNC	-	-	21.66 ± 0.77	-	-
Fe-GCNC	-	17.76 ± 0.36	3.23 ± 0.11	-	-
Pd-Pt/GCNC	2.11 ± 0.13	-	3.56 ± 0.10	3.03 ± 0.18	0.38 ± 0.03
Pd-Pt/GCNC5× used	0.24 ± 0.01	-	3.16 ± 0.16	3.54 ± 0.22	0.42 ± 0.02
Pd-Pt/Ni-GCNC	-	-	21.07 ± 0.55	2.68 ± 0.42	0.45 ± 0.01
Pd-Pt/Ni-GCNC5× used	-	-	12.72 ± 0.86	1.41 ± 0.11	0.15 ± 0.01
Pd-Pt/Fe-GCNC	-	13.69 ± 4.27	3.19 ± 0.25	2.38 ± 0.28	0.28 ± 0.03
Pd-Pt/Fe-GCNC5× used	-	9.20 ± 0.89	3.22 ± 0.12	1.64 ± 0.16	0.20 ± 0.02

## Supplementary Materials

The following supporting information can be downloaded at: https://www.mdpi.com/article/10.3390/ijms231810514/s1.
