# Supplementary material for: Development of Palladium and Platinum Decorated Granulated Carbon Nanocomposites for Catalytic Chlorate Elimination
Source: Int J Mol Sci. 2022 Sep 10;23(18):10514. doi: 10.3390/ijms231810514 (PMC9500763; doi:10.3390/ijms231810514)
Supplement: Supplementary file 1 [file ijms-23-10514-s001.zip › ijms-1911705-supplementary.pdf]

Development of Palladium and Platinum Decorated Granulated Carbon Nanocomposites for Catalytic Chlorate Elimination

Emőke Sikora [1,*], Gábor Muránszky [1], Ferenc Kristály [2], Béla Fiser [1,3,4], László Farkas [5], Béla Viskolcz [1,3] and László Vanyorek [1,*]

1 Institute of Chemistry, University of Miskolc, H-3515 Miskolc-Egyetemváros, Hungary
2 Institute of Mineralogy and Geology, University of Miskolc, H-3515 Miskolc-Egyetemváros, Hungary
3 Higher Education and Industrial Cooperation Centre, University of Miskolc, H-3515 Miskolc-Egyetemváros, Hungary
4 Ferenc Rakoczi II Transcarpathian Hungarian College of Higher Education, 90200 Beregszász, Transcarpathia, Ukraine
5 BorsodChem Ltd, Bolyai tér 1., H-3700 Kazincbarcika, Hungary
* Correspondence: kemsik@uni-miskolc.hu (E.S.); kemvanyi@uni-miskolc.hu (L.V.)

SUPPLEMENTARY MATERIALS

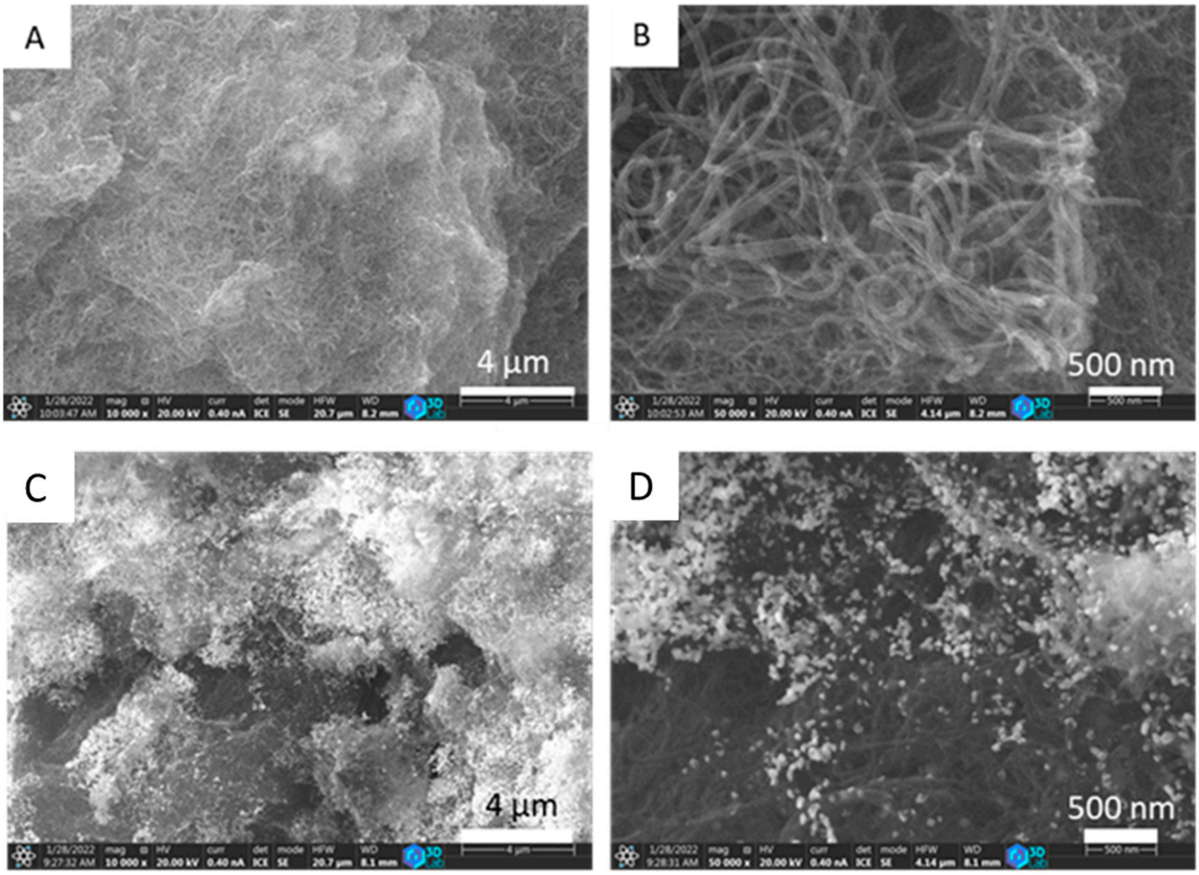

Figure S1. SEM images of the prepared calcium containing granulated carbon nanocomposite (GCNC) support (A, B) and the corresponding Pd-Pt/GCNC (C, D) catalyst.

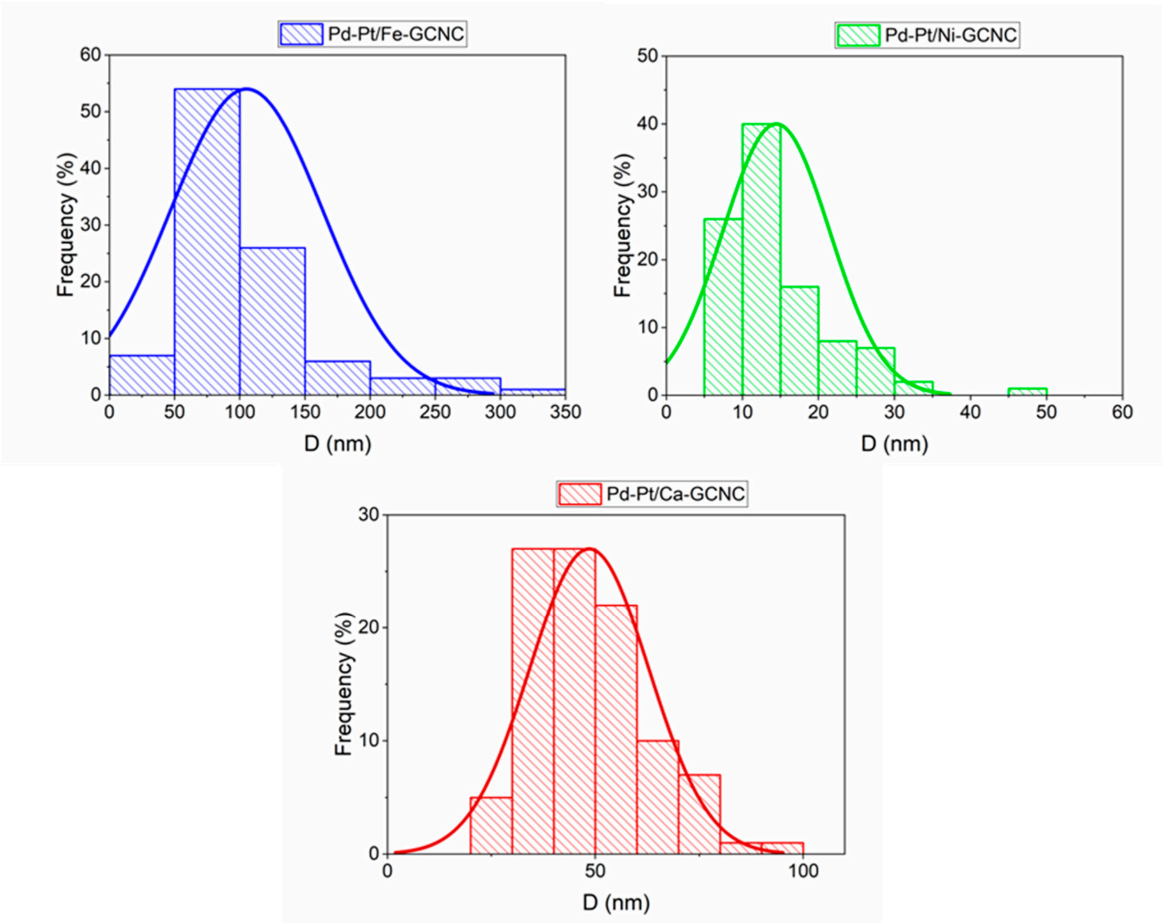

Figure S2. Size distribution of the nanoparticles in the samples based on the SEM images.
